# Alzheimer's Disease and Vascular Deficiency: Lessons from Imaging Studies and Down Syndrome

**DOI:** 10.1155/2012/929734

**Published:** 2012-02-15

**Authors:** Arlene Reed-Cossairt, Xiongwei Zhu, Hyoung-Gon Lee, Charles Reed, George Perry, Robert B. Petersen

**Affiliations:** ^1^Department of Special Education and Early Childhood Studies, Boise State University, 9921 W. Edna, Boise, ID 83704, USA; ^2^Department of Pathology, Case Western Reserve University, Cleveland, OH, USA; ^3^Department of Health and Epidemiology, Southwest District Health, Nampa, ID 83605, USA; ^4^UTSA Neurosciences Institute of Department of Biology, University of Texas at San Antonio, San Antonio, TX, USA; ^5^Department of Neuroscience, Case Western Reserve University, Cleveland, OH, USA; ^6^Department of Neurology, Case Western Reserve University, Cleveland, OH, USA

## Abstract

Down syndrome (DS) individuals are at high risk for developing Alzheimer's disease (AD) and consequently provide a unique opportunity to examine the factors leading to the onset of AD. This paper focuses on the neglected vascular parallels between AD and DS that can readily be examined in DS. Several recent AD studies provide evidence that internal jugular vein (IJV) reflux may result in white matter lesions and a 30% decrease in cerebrospinal fluid (CSF) clearance of amyloid-**β**. At the same time, studies analyzing the synthesis of amyloid-**β** in DS showed greater than expected amounts of A**β** than would be predicted by the increase in gene dosage, perhaps due to slower clearance. These studies are discussed along with the possibility that the venous and CSF dysfunction found in AD patients may be present early in life in persons with DS, leaving them particularly vulnerable to early onset AD. Studying IJV function in DS provides an opportunity to understand the role of vascular function in the initiation of AD.

## 1. Introduction

The brains of most individuals with Down syndrome (DS) who are over 40 years old will have sufficient neuropathology for a postmortem diagnosis of Alzheimer's disease (AD) and provide an ideal population to examine novel ideas about the causation of AD. DS is a very complex genetic disorder that produces detrimental changes to many organ systems. The mechanism(s) by which the extra copy of chromosome 21 or parts thereof produce these changes is largely unknown [1]. The majority of research and therapeutic efforts to date have focused on the diagnosis and surgical correction of major heart defects associated with DS, once a major killer of children with the condition. With the cardiovascular defects surgically corrected, the average lifespan of persons with DS has increased significantly. Consequently, the spectrum of threats to persons with DS include childhood illnesses early in life and the development of AD later in life.

 Increasing evidence indicates that AD is a neurovascular disease, with macrovascular events such as heart attack and stroke causing sustained hypoxia preceding disease onset [2], although some cases of AD lack a vascular component. DS typically presents with many vascular defects that are rarely seen in the general population (Table 1).

 Microvascular dysfunction also appears to play a significant role in AD onset and progression [5], and the vascular endothelium may be dysfunctional in DS. Recent studies describe severe dysfunction in the endothelial system in DS [6], including significantly lower levels of endothelial progenitor cells that are necessary for vascular regrowth and repair after injury [6]. This may result from the early occurrence of oxidative stress in DS [7], which has been linked to defects in vascular epithelium [8]. Consequently, when a patient with DS has an accident or event involving vascular injury, that vascular system will not repair itself as quickly or as effectively as a person without DS. Additionally, AD risk is increased by head trauma or stroke [2], and normal repair and regeneration normally decline with age.

 Finally, a recent review of AD research by Humpel [9] proposes a much larger role for the vascular system as a whole in the development of AD and details the impact of chronic mild cerebrovascular dysfunction on the disease. Humpel proposes that the onset of AD is preceded by chronic exposure to the cardiovascular risks over many years, including hyperhomocysteinemia, hypercholesterolemia, and type-2 diabetes, all of which cause damage to the cerebrovascular system. Amyloid-*β* (A*β*) deposition may be a secondary consequence of these ongoing vascular insults. DS often presents with these risk factors, in addition to the vascular defects and endothelial dysfunction previously described [10–12]. Therefore, the DS population should figure prominently in studies on the onset and progression of AD.

 Most of the research on DS and AD focuses on genes related to the production of A*β*. Genetic studies have confirmed that the amyloid-*β* protein precursor (A*β*PP) gene and genes associated with A*β* production are located on chromosome 21 [13]. Persons with AD also exhibit trisomy 21 in various cell types, such as skin fibroblasts, peripheral blood lymphocytes, and brain neurons, although the relevance of this to disease onset is yet to be established [14]. The overexpression of A*β* from the extra copy of chromosome 21 has been posited as the primary driver of AD in DS, with overproduction of A*β* being responsible for its deposition in the brain. Several recent imaging studies provide insight into the very early stages of AD and suggest that vascular changes figure prominently in the development and possibly initiation of AD, as in DS.

## 2. White Matter Changes in Alzheimer Disease

White matter changes have been found even in the preclinical stages of AD. Gold and colleagues [15] analyzed white matter changes in women at high risk for developing AD (those with at least one APOE4 allele and a family history of dementia) and compared them to women at low risk (no risk factors). Women at high risk of developing AD showed several patterns of white matter changes not present in healthy controls, including in the direct and indirect connections to the median temporal lobes, as measured by diffusion tensor imaging [15]. Additionally, Sanz-Arigita et al. [16] used fMRI to examine resting state brain function in persons diagnosed with mild AD as compared to healthy controls. Here, brains of persons with mild AD showed regional changes in function in the frontal lobes, including increased synchronization, and the caudal areas had decreased synchronization, which may be indirectly linked to white matter changes. Conversely the occipital and parietal lobes were unaffected. Sanz-Arigita et al. conclude that there may be a global loss of long distance connections between the frontal and caudal regions [16]. Interestingly, changes in the “presymptomatic individuals” [15] involved connectivity largely in the frontal tracts, while individuals with mild AD had more global changes involving long distance connectivity [16]. Further studies are required to determine whether these results accurately indicate the pattern of disease progression. While the two studies used different patient populations and imaging techniques, both support a model of progressive change in white matter function very early in disease progression.

## 3. Impaired Clearance of Amyloid-***β***

The studies described above provide imaging not previously available and are indicative of changes in the white matter in AD, but do not address the mechanism driving these changes. Overproduction of A*β* is thought to be the major source of damage to white matter in AD [2]. However, a recent study by Mawuenyega et al. provides an alternative possibility for the accumulation of A*β* in the brain [17]. In this study, the researchers used mass spectroscopy to longitudinally measure the level of A*β* in cerebrospinal fluid (CSF), as well as the clearance and production rates of A*β*. Importantly, it should be noted that CSF clearance is largely through the white matter and is negligible in gray matter [18]. Mawuenyega et al. measured clearance and production rates for A*β*_42_ and A*β*_40_ for 36 hours in 12 patients with late-onset AD, as compared to healthy controls. The AD group had a 30% slower A*β* clearance rate than the controls, although no difference in average production rates was seen between the AD group and healthy controls [17].

 CSF clearance may be an important disease marker in AD. Ott and colleagues examined increased ventricular volume as a biomarker for impaired CSF clearance [19]. They studied the relationship between ventricular volume and the AD-related biomarkers A*β*, tau, and phosphorylated tau in controls, individuals with mild cognitive impairment, and individuals with AD, taking ApoE genotype into account. Here, ventricular volume was inversely related to A*β* levels for ApoE4 controls and to tau levels in AD patients [19], although the mechanism underlying the ApoE4 effect on ventricular volume is unclear. Wastyn et al. described the protective effects of daily consumption of caffeine with regard to AD [20], which appears to be due to caffeine's affect on the CSF system and resulting clearance of various toxins, including A*β* and tau [20].

 Taken together, these studies indicate that the onset of AD may not be due to an overproduction of A*β*, but rather by impaired CSF drainage and flow. Therefore, further studies are needed to determine the mechanism governing CSF drainage and flow. CSF is produced in the choroid plexus and is reabsorbed into the bloodstream via the arachnoid villi and venous sinuses [21]. Changes in CSF production, pressure, and flow rates are affected by external forces causing inflammation or leakage, including traumatic brain injury, infection, tumors, or lumbar punctures [21]. The result of impaired clearance may be specific to AD, reflecting the extracellular presence of A*β* in contrast to tau, alpha synuclein, and ubiquitin, and suggests that the role of A*β* in disease initiation and progression results from production/secretion of A*β* rather than release of A*β* following cell death.

 The CSF system may also be influenced by changes in vascular flow and pressure of the venous systems near the brain. Alteration of homeostasis between the CSF and the vascular system may play an important role in the development of AD; dysfunction in the vascular systems involved with CSF may decrease CSF clearance from the brain, thereby increasing A*β* in the brain resulting in disease onset. One such age-related change in the vascular system is jugular venous reflux, which can lead to decreased cerebral perfusion pressure [22]. The internal jugular vein provides the majority of the drainage pathway for cerebral venous drainage. Jugular venous reflux results from pressure beyond the competence of the IJV valves and consequent increased backpressure limiting cerebral perfusion pressure. Jugular venous reflux is linked to a variety of other neurological disorders, including transient global amnesia, transient monocular blindness, multiple sclerosis, exertional headaches, and idiopathic intracranial hypertension [22], all of which may be linked to increased oxidative stress.

## 4. Internal Jugular Reflux Increases with Age

Vascular events are a prominent risk factor for the development of AD [2]. Chung and colleagues performed color-coded duplex sonography on the internal jugular veins (IJVs) of 349 subjects ranging in age from 55.6 to 89 years old [23]. These subjects comprised a large, healthy population, with age being the main variable among them. Overall IJV function changed with increasing age, although this occurred particularly in the left IJV, including increased lumen area, increased jugular venous reflux, and slower velocity. These findings are consistent with decreased left IJV outflow with aging [23].

## 5. White Matter Changes with Jugular Reflux

Changes in white matter often occur with the onset of AD [15, 16]. In a recent MRI and ultrasound study, white matter changes were also found to be associated with IJV reflux [24]. Here, MRI and ultrasound were used to analyze the brains and IJVs, respectively, of 97 individuals ranging in age from 55 to 90 years old. The ultrasound results were grouped into three categories of jugular venous reflux: none, mild, and severe. Persons with severe jugular venous reflux had more white matter changes than either the mild or no reflux groups, particularly in caudal brain regions. Further, whole brain white matter changes were more prominent in persons greater than 75 years old that had severe venous reflux [24], consistent with previous findings.

 Taken together, these studies provide a potential mechanism by which IJV function affects CSF flow, leading to the development of AD. Specifically, IJV function declines with age, resulting in reflux, slower velocity, and decreased venous outflow. This decreased flow produces changes in venous pressures, which then alters pressure in the CSF system. The CSF system decreases outflow from the brain to restore homeostatic pressure in the brain. As a consequence, A*β* begins to accumulate within the brain instead of being cleared via the CSF. Increased A*β* accumulations lead to damage to white matter, beginning with the temporal lobes and expanding to frontal and caudal regions, perhaps ultimately leading to clinical features associated with AD.

## 6. Relevance to Down Syndrome

The CSF clearance study by Mawuenyega et al. [17] provides evidence that, in the general population, clearance may be more important in the etiology of AD than is production of A*β*. Currently, no comparable studies in a DS population have been conducted, although several studies provide indirect evidence that A*β* clearance may be a factor in DS. Gyure and colleagues found that serum levels of A*β* are 200–300% higher in DS individuals as compared to controls [25], possibly due to overproduction of A*β*. Wolvetang and coworkers examined A*β* production in relation to the predicted effect of gene dosage and found that A*β* expression is 3-4 times higher in DS individuals, rather than 1.5 times higher as would be predicted due to the extra chromosome 21. Wolvetang et al. concluded that an additional transcriptional regulator of A*β* also located on chromosome 21 may cause overexpression of A*β* protein in DS [26]. Finally, Choj et al. examined levels of A*β*PP with increasing age in a mouse model of DS [27] and determined that DS mice expressed the same level of A*β*PP as controls at 4 months of age. However, by 10 months of age, the DS mice exhibited increased levels of A*β*PP [27], although A*β*_40_ and A*β*_42_ were not increased. Choj et al. concluded that the changes in A*β*PP levels are due to “multiple mechanisms of regulation” [27]. Taken together, overproduction alone could not fully explain increased A*β* levels. As reviewed in Wiseman et al. [28], a number of additional genes on chromosome 21 have been implicated in the development of AD in DS individuals, including those involved in tau hyperphosphorylation (e.g., DYRK1A).

## 7. Down Syndrome and Alzheimer Disease: Vascular Risks

Given the large number of known vascular problems present in individuals with DS, it is possible that the vascular system associated with CSF clearance, particularly the IJVs studied by Chung et al. [23, 24], could be impaired in DS and that this impairment would likely begin early in life. Chronic, yet mild, dysfunction of the IJV beginning early in life, along with resulting impairment in CSF clearance, would leave persons with DS particularly vulnerable to the buildup of A*β* in the brain, which may be exacerbated by the overproduction of A*β*PP due to increased gene dosage.

 Together, the studies described above addressed very specific questions relating to gene expression and protein production, but did not examine CSF clearance. Based on the CSF clearance study, one should question whether increased production of A*β* is the only cause of high levels of A*β* in DS. Perhaps persons with DS have severe CSF clearance issues along with increased production. Or could it be due to complications from cardiovascular problems seen in early life and not fully corrected by surgical treatment?

 The role of IJV reflux and CSF clearance of A*β* in the development of AD in the DS population is currently unknown. Determining whether these two conditions occur in the DS population would help to clarify the role of overproduction of A*β* versus the role of vascular defects and dysfunction in the development of AD for persons with DS.

 Replicating the studies described above in a DS population would provide answers to several key questions, including the following.

**CSF Clearance of A*β* in Down Syndrome**

- Is the rate of A*β* production increased in DS relative to healthy controls, to AD patients?
- Do adult patients with DS and AD exhibit decreased CSF clearance of A*β*?
- Do adult patients with DS, but not AD, exhibit decreased CSF clearance of A*β*?
- Do children with DS exhibit decreased CSF clearance of A*β*?

**IJV Function and Resulting White Matter Changes in Down Syndrome**

- How do the IJVs function in adults with DS, as compared to healthy controls? Compared to those with AD?
- Does IJV function deteriorate more quickly in DS than in healthy controls and/or those with AD?
- Do persons with DS present with jugular reflux? If so, do they also present with changes in white matter consistent with the patients previously studied?
- Do children with DS present with IJV dysfunction. That is, at what age does jugular reflux begin?

Taken together, the studies outlined above suggest a temporal sequence of events beginning with increased oxidative stress, an early feature of AD. Chronic oxidative stress, in turn, may lead to decreased vascular function and ultimately results in increased A*β* deposition. The increased expression of A*β*PP and A*β* appears to be a compensatory response to stress and deposition may simply reflect the failure of this response to alleviate chronic stress in the context of decreased clearance. Studies of the DS population will aid in clarifying these interactions, perhaps elucidating a potential point of intervention in the development of AD pathology in these individuals.

## Figures and Tables

**Table 1:** Vascular defects in Down syndrome: birth defects and prenatal vascular findings [3, 4].

Birth defects	Prenatal vascular findings
Cardiac defects (VSD and ASD) (found in 50% of persons with DS)	Reverse flow in the ductus venosus (90% of all DS fetuses)
Intrahepatic venous anomalies	Placental hypovascularity (100%)
Pelvic vasculature malformations	Intrathoracic vascular lesions (more rare, probably leads to fetal demise)
Pulmonary vein obstruction	Umbilicoportal vascular anomalies (most common fetal defect in DS)
Aortopulmonary collateral arteries	
Anomalous aortic arch arteries	
Aberrant right subclavian artery (found in 20–40% of persons with DS)	
Moyamoya disease	
Arterial dysplasia	
Thrombosis of the venous sinuses	

*Birth defects: *anomalies found at birth or later in life. May be found due to symptoms, or may be found incidentally. Some can also be found via pre-natal ultrasound, such as the cardiac defects, and aberrant right subclavian artery.

*Prenatal vascular findings:* anomalies found via pre-natal ultrasound, either in a research or clinical setting. Many of these anomalies will resolve at birth.

VSD: ventricular septal defect; ASD: atrial septal defect.
